# Prevalence of Antibiotic-Resistant *Escherichia coli* in Drinking Water Sources in Hangzhou City

**DOI:** 10.3389/fmicb.2017.01133

**Published:** 2017-06-16

**Authors:** Zhaojun Chen, Daojun Yu, Songzhe He, Hui Ye, Lei Zhang, Yanping Wen, Wenhui Zhang, Liping Shu, Shuchang Chen

**Affiliations:** ^1^Department of Environmental and Occupational Health, Hangzhou Center for Disease Control and PreventionHangzhou, China; ^2^Department of Clinical Laboratory, Hangzhou First People's HospitalHangzhou, China; ^3^Department of Clinical Laboratory, Guilin Medical University Affiliated HospitalGuilin, China; ^4^Department of Automatic Monitoring, Hangzhou Environmental Monitoring CenterHangzhou, China; ^5^Dean's Office, Hangzhou Prevention and Treatment Center for Occupational DiseasesHangzhou, China; ^6^Department of Microbiology Laboratory, Hangzhou Center for Disease Control and PreventionHangzhou, China

**Keywords:** antibiotic resistance, drinking water source, *Escherichia coli*, water quality, *tet* genes

## Abstract

This study investigated the distribution of antibiotic resistant *Escherichia coli* (*E. coli*) and examined the possible relationship between water quality parameters and antibiotic resistance from two different drinking water sources (the Qiantang River and the Dongtiao Stream) in Hangzhou city of China. *E. coli* isolates were tested for their susceptibility to 18 antibiotics. Most of the isolates were resistant to tetracycline (TE), followed by ampicillin (AM), piperacillin (PIP), trimethoprim/sulfamethoxazole (SXT), and chloramphenicol (C). The antibiotic resistance rate of *E. coli* isolates from two water sources was similar; For *E. coli* isolates from the Qiantang River, their antibiotic resistance rates decreased from up- to downstream. Seasonally, the dry and wet season had little impact on antibiotic resistance. Spearman's rank correlation revealed significant correlation between resistance to TE and phenicols or ciprofloxacin (CIP), as well as quinolones (ciprofloxacin and levofloxacin) and cephalosporins or gentamicin (GM). Pearson's chi-square tests found certain water parameters such as nutrient concentration were strongly associated with resistance to some of the antibiotics. In addition, *tet* genes were detected from all 82 TE-resistant *E. coli* isolates, and most of the isolates (81.87%) contained multiple *tet* genes, which displayed 14 different combinations. Collectively, this study provided baseline data on antibiotic resistance of drinking water sources in Hangzhou city, which indicates drinking water sources could be the reservoir of antibiotic resistance, potentially presenting a public health risk.

## Introduction

There is a growing concern regarding the occurrence of antibiotic resistant bacteria (ARB) and antibiotic resistance genes (ARGs) in aquatic environments (Kummerer, 2009; Diwan et al., 2010). As the main receptacle for pollution from industry, agriculture, or domestic life, aquatic environment provides an ideal setting for the acquisition and dissemination of antibiotic resistance (Pereira et al., 2013; Marti et al., 2014). Natural bodies of water, commonly used for irrigation, aquaculture, or recreation activities, are closely associated with human life. Direct or indirect contact with water (for drinking, or recreational use) contaminated by ARB could harm and infecte the human population with antibiotic resistant pathogens, and/or ARGs carried by bacteria may transfer to microorganisms in humans as a consequence of horizontal gene transfer (Chen et al., 2011; Heuer et al., 2011; Ribeiro et al., 2012; Jiang et al., 2013). Such events would undermine our ability to prevent and control disease, and thus expose a great threat to public health.

Most of the studies focusing on antibiotic resistance are done in aquatic environments with serious pollution or waters that have been strongly influenced by anthropogenic activities, such as agricultural watershed and rivers near wastewater treatment plant outflows (Maal-Bared et al., 2013; Middleton and Salierno, 2013; Zhang et al., 2014). However, the presence and distribution of antibiotic resistance in a special aquatic environment, such as a drinking water source, is always neglected due to strict legal protection and less anthropogenic activities. According to literatures, the context of antibiotic resistance in drinking water sources is of serious grave concern (Jiang et al., 2013; Flores Ribeiro et al., 2014; Guo et al., 2014; Machado and Bordalo, 2014; Mohanta and Goel, 2014). For example, high antibiotic resistance rates of 72 and 59% were found in a water source in Guinea-Bissau (West Africa) during both the dry and wet seasons, (Machado and Bordalo, 2014). High antibiotic resistance levels were also observed in the source waters of the Huangpu River of China (Jiang et al., 2013). Considering the source water is directly related with human activity and health, understanding the prevalence of antibiotic resistance in human drinking water sources is of great importance.

Hangzhou, the capital of Zhejiang Province in China, is one of the most economically developed cities in China. The Qiantang River and the Dongtiao Stream both serve this city of 1.96 million residents as the drinking water sources. Unfortunately, the Qiantang River and the Dongtiao Stream both face serious pollution from the upstream cities' discharge. For instance, the Fuchun River is upstream to the Qiantang River, with many potential pollution sources existing in its downstream, such as wastewater treatment plants (WWTPs), large and small industrial plants, sand excavation operation sites, and livestock farmland. With this, various pollutants including antibiotics, pesticides, and insecticides likely flow into the Qiantang River promoting the emergence and spread of antibiotic resistance in drinking water sources (Zhao and Dang, 2011). Nevertheless, limited information about antibiotic resistance in these water sources has been made available.

*Escherichia coli* (*E. coli*) is an opportunistic pathogen that can survive well in aquatic environments, and *E. coli* is highly adept at horizontal gene transfer, which is deemed as the vector for antibiotic resistance dissemination (Maal-Bared et al., 2013; Pereira et al., 2013). For the present study, as the situation of drinking water source can be best represented by water collected from the intake sites of water plants, we collected samples from five intake sites of water plants whose water sources are the Qiangtang River and the Dongtiao Stream. We investigated the presence of antibiotic resistant *E. coli* from samples to 18 antibiotics that are commonly used in clinical and farming practices. We further examined the effects of water quality parameters and seasonal variations on antibiotic resistance. The working hypothesis was that (1) *E. coli* isolates from different water sources and different seasons may have different antibiotic resistance levels; (2) With good protection and less new pollution, the antibiotic resistance levels of *E. coli* may be decreased; (3) Some water quality parameters could have an impact on the levels of antibiotic resistance.

## Materials and methods

### Sampling-sites description and sample collection

**Figure 1:**
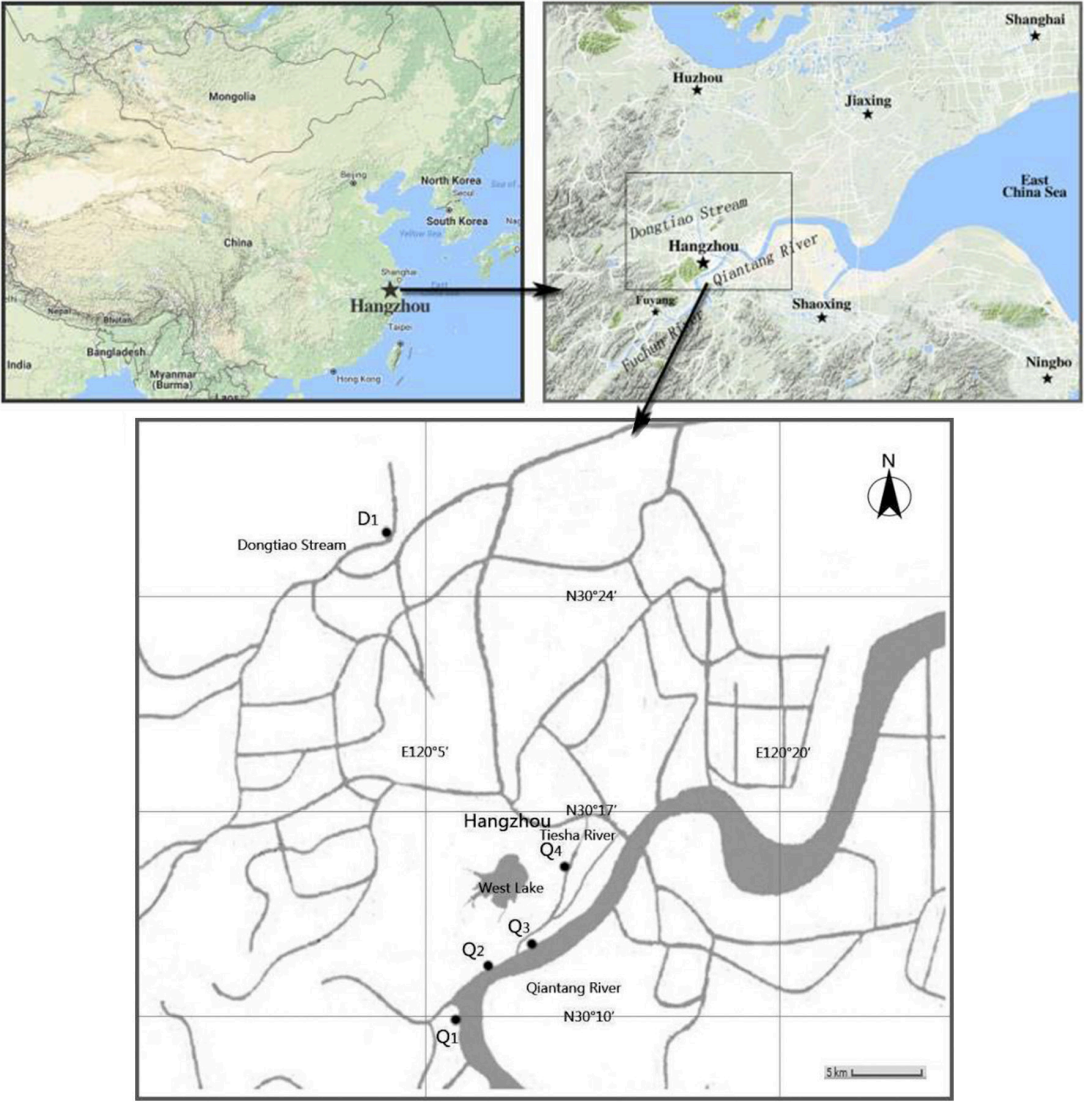
Geographic locations for sampling sites in the Qiantang River and the Dongtiao Stream.

The Qiantang River (Q) supplies water to three water plants while the Dongtiao Stream (D) is the source for one additional water plant, respectively. Sampling sites (Q1, Q2, Q3, and D1) were selected at the corresponding water intakes of the four water plants. The Tiesha River (site Q4) is one tributary of the Qiantang River, which serves as a water source with an urban backup. All of the sampling sites (Figure 1) are routinely used for water quality monitoring by the local environmental authorities.

Triplicate samples were collected on April 3, 2014 (dry season) and July 4, 2014 (wet season). These samples were then separated into two parts: (1) 500 ml water samples were collected using sterile glass bottles for the evaluation of microbiological parameters and the isolation of *E. coli*; (2) 5 L water samples were obtained using plastic barrels for the analysis of physicochemical parameters of the water. All samples were preserved in cold boxes, transported to the laboratory within 4 h and maintained at 4°C until use.

### Water quality data

All water quality parameters were measured according to Chinese National Standard Methods. Briefly, water temperature (Thermometer method), pH (Glass electrode method), and dissolved oxygen (Electrochemical probe method) were measured in situ using a digital display thermometer (Ruiming, Changzhou, China), a pH meter (METTLER, Switzerland) and an YSI-58 instrument (Yellow Spring, USA) respectively. Other water quality parameters analyzed in the lab were ammonia nitrogen, total phosphorus, total nitrogen, nitrate-N, biological oxygen demand, chemical oxygen demand, fecal coliforms, and *E. coli*. Ammonia nitrogen (Nessler's reagent spectrophotometry method) and total phosphorus (Ammonium molybdate spectrophotometric method) were measured using the visible spectrophotometer (UNICO, Shanghai, China). Total nitrogen (Potassium persulfate digestion UV spectrophotometric method nitrate-N) and nitrate-N (Spectrophotometric method with phenol disulfonic acid method) were measured using a double beam UV-visible spectrophotometer (PERSEE, Beijing, China). Biological oxygen demand was measured using the dilution and seeding method. COD was measured using the dichromate method. Fecal coliforms were measured using the manifold zymotechnics method. And *E. coli* was measured using the membrane filter method.

### Isolation of *E. coli* and antibiotic susceptibility test

The number of *E. coli* was determined in water samples by membrane filtration method (Talukdar et al., 2013). Briefly, a 50 ml aliquot of water sample was filtered through a 0.22 μm membrane filter (Millipore, Carrightwohill, IRL). The membranes were placed on *E. coli*-Coliforms Chromogenic Mediums (Nissui, Qingdao, China) and incubated at 37°C for 24 h. After incubation, blue colonies represented *E. coli*. Typical *E. coli* were picked and purified on the MacConkey Agar (Nissui, Qingdao, China), and then they were confirmed using the automated identification and susceptibility testing system (BD Phoenix NMIC/IDNMIC/ID-4 panel, Sparks, USA) Confirmed isolates were stored at −80°C in tryptic soy broth with 20% (vol/vol) glycerol.

Antibiotic susceptibility testing for confirmed *E. coli* was conducted using the automated identification and susceptibility testing system (BD Phoenix NMIC/IDNMIC/ID-4 panel, Sparks, USA). Eighteen antibiotics were contained: amikacin (30 μg), gentamicin (10 μg), imipenem (10 μg), meropenem(30 μg), cefazolin (30 μg), ceftazidime (30 μg), cefotaxime (30 μg), cefepime (30 μg), aztreonam (30 μg), ampicillin (10 μg), piperacillin (100 μg), amoxicillin/clavulanate (20/10 μg), ampicillin/sulbactam (10/10 μg), chloramphenicol (30 μg), ciprofloxacin (5 μg), levofloxacin (5 μg), tetracycline (30 μg). “Sensitive” “intermediate resistant” or “resistant” patterns of *E. coli* were judged using antibiotic minimum inhibitory concentration(MIC) provided by the guidelines of the Clinical and Laboratory Standards Institute (CLSI). *E. coli* strain ATCC 25922 was sensitive to all the antibiotics tested and so was used as the quality control strain. All isolates showing “resistant” or “intermediate resistant” patterns were classified as “resistant,” whereas all other isolates were classified as “sensitive”. *E. coli* isolates resistant to three or more classes of antibiotics were multiple resistance *E. coli* isolates, which were classified as R3-6 subpopulation. And isolates resistant to one or two classes of antibiotics were classified as R1-2 subpopulation.

The antibiotic resistance index (ARI) is used for analyzing the prevalence of *E. coli* isolated from drinking water sources. ARI was determined using the following formula: ARI = a/(b × c), where “a” is the total antibiotic resistance score of all *E. coli* from specified location, “b” is the number of tested antibiotics, and “c” is the number of *E. coli* isolates from specified location (Mohanta and Goel, 2014; Zhang et al., 2014).

### Detection of tetracycline resistance genes

Genomic DNA was extracted from *E. coli* isolates by the boiling method (Dei-Tutuwa and Rahman, 2014). Briefly, colonies of *E. coli* were suspended in 100 μl of distilled water and heated at 100°C for 10 min followed by cooling for 10 min. The lysed cell suspension was centrifuged at 12,000 g for 10 min, and the recovered supernatant was frozen at −20°C until use.

PCR assays were used to determine the presence of tetracycline resistance genes in tetracycline-resistant *E. coli*. We designed the primers using Primer Primiers 5.0 software. Primers with the best specificity and similar melting temperatures (T_m_) were selected based on a blast sequence comparison. The primers were synthesized by Lift Technology, and their sequences are listed in Table 1.

**Table 1:** Primer sets for the detection of *tetA, tetC, tetD, tetE*, and *tetM* used in this study.

**Target gene**	**Primer sequence (5′-3′)**	**Product size(bp)**	**References**
*tetA*	F: GCTACATCCTGCTTGCCTTC	210	Ng et al., 2001
	R: CATAGATCGCCGTGAAGAGG		
*tetC*	F: CTTGAGAGCCTTCAACCCAG	418	Ng et al., 2001
	R: ATGGTCGTCATCTACCTGCC		
*tetD*	F: CGGCAATACTGAATGCCTGC	461	This study
	R: AGGACCGGATACACCATCCA		
*tetE*	F: TGAACCGCACTGTGATGATG	255	This study
	R: CGTAGTCCAGTGTTGCACCT		
*tetM*	F: GCAATTCTACTGATTTCTGC	456	This study
	R: CTGTTTGATTACAATTTCCGC		

Amplification of the DNA was performed in a PCR apparatus with TaKaRa Ex Taq buffer (TaKaRa, Japan). A positive control, a negative control and a blank control were employed in this assay.

PCR products of tetracycline resistance genes obtained from the resistant isolates were cloned, sequenced, and searched against GenBank using the BLAST alignment tool (http://www.ncbi.nlm.nih.gov/blast/).

### Statistical analyses

All statistical analyses were conducted using SPSS15.0. Rank-sum tests were performed to evaluate the difference of water quality parameters in different drinking water sources or seasons. Chi-square tests were used to determine the effect of different water quality parameters or different seasons on the resistance patterns of *E. coli*. To describe the relationships between antibiotic resistance rates and water quality parameters, Spearman's rank correlations were used for each antibiotic. Person's chi-square tests were used to evaluate the association between different antibiotic resistance phenotypes of *E. coli* isolates from drinking water sources.

## Results

### Water quality of drinking water sources

As shown in Table 2, some physicochemical and bacteriological parameters of water samples were measured, including temperature, dissolved oxygen (DO), pH, chemical oxygen demand (COD), Nitrate -N(${\text{N}0}_{3}^{-1}$-N), and fecal coliforms. Except for DO and fecal coliforms at site Q4 in the wet season, all values of water quality parameters tested met Surface Water Environmental Quality Standard for grade III (GB3838-2002), which was the standard for evaluating the water quality of centralized drinking water source supplies in China. It should be noted that, however, the values of total phosphorus (TP) and fecal coliforms at some sampling sites were close to the upper limit (0.2 mg/l and 1^*^10^4^ CFU/l, respectively), and the values of total nitrogen (TN) were high in all samples which ranged from 1.91 to 3.54 mg/l, indicating that some degree of organic pollution and fecal pollution existed in drinking water sources.

**Table 2:** Water quality parameters of the Qiantang River and the Dongtiao Stream between the dry and wet seasons.

**Sampling periods**	**Sources**	**Sites**	**PH**	**Water temp. (°C)**	**DO (mg/l)**	**Ammonia nitrogen (mg/l)**	**Total phosphorus (mg/l)**	Total nitrogen^*^ (mg/l)	Nitrate -N^*^ (mg/l)	**BOD_5_ (mg of O_2_/l)**	COD^*^ (mg of O_2_/l)	Fecal coliforms^*^ (CFU/100 ml)	E. coli^*^ (CFU/100ml)
Dry season	Qiantang River	Q1	7.47	16.7	6.24	0.465	0.130	3.42	2.36	0.5	2.22	110	28
		Q2	7.43	16.4	6.12	0.471	0.152	3.54	2.31	0.8	2.71	460	29
		Q3	7.41	16.2	6.45	0.620	0.140	3.31	2.37	0.6	2.44	1,100	28
		Q4	7.55	18.3	6.74	0.226	0.069	3.28	2.47	1.2	4.00	2,000	262
	Dongtiao Stream	D1	7.66	14.2	7.32	0.546	0.104	3.49	2.62	1.7	2.90	940	272
Wet season	Qiantang River	Q1	7.36	22.5	7.28	0.408	0.112	2.33	1.61	1.2	1.14	5,400	388
		Q2	7.43	22.6	7.15	0.347	0.125	2.71	1.64	1.7	1.22	2,400	286
		Q3	7.42	22.6	7.23	0.377	0.128	2.39	1.62	1.5	1.38	9,200	544
		Q4	7.40	22.7	**3.95**	0.303	0.074	1.91	1.59	0.9	1.00	**35,000**	1,560
	Dongtiao Stream	D1	7.31	21.2	5.70	0.358	0.091	3.93	1.67	2.0	2.10	2,800	1,620

* Statistically significant differences were found between the dry and wet seasons;

*DO, dissolved oxygen; Water temp., water temperature. Bold: the values are higher than standards*.

Rank-sum tests were used to determine the statistical significance of organic pollution and fecal pollution in different drinking water sources and seasons. The results showed significant seasonal differences for ${\text{N}0}_{3}^{-1}$-N, TN, COD, fecal coliforms, and *E. coli*, with *p*-values of 0.009, 0.009, 0.021, 0.009, and 0.009, respectively, no significant difference was found between the two drinking water sources for organic pollution and fecal pollution. These data suggest that the level of organic pollution was higher in the dry season, while the pollution with fecal materials was more serious in the wet season.

### The prevalence of antibiotic resistant *E. coli* and antibiotic resistance profiles in drinking water sources

**Figure 2:**
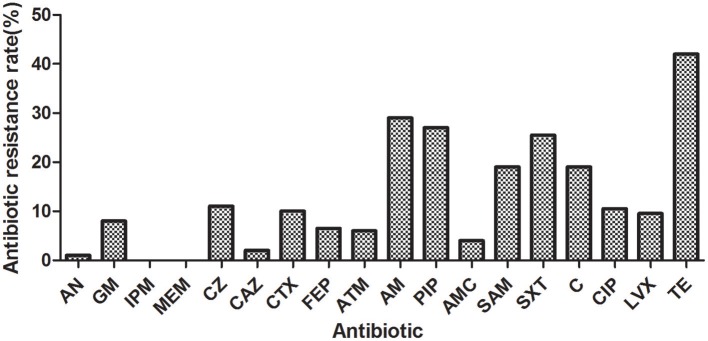
Antibiotic resistance rates of the *E. coli* isolated from drinking water sources (the Qiantang River and the Dongtiao Stream). AN, amikacin; GM: gentamicin; IPM, imipenem; MEM, meropenem; CZ, cefazolin; CAZ, ceftazidime; CTX, cefotaxime; FEP, cefepime; ATM, aztreonam; AMP, ampicillin; PIP, piperacillin; AMC, amoxicillin/clavulanate; SAM, ampicillin/sulbactam; C, chloramphenicol; CIP, ciprofloxacin; LVX, levofloxacin; TE, tetracycline.

A total of 200 *E. coli* isolated from drinking water sources were examined. Among them, 99 isolates (49.50%) were resistant to at least one of the 18 antibiotics tested, and the antibiotic resistance index was 0.13. As shown in Figure 2, the most frequently resisted antibiotic was tetracycline (TE) (42.00%), followed by ampicillin (AM) (29.00%), piperacillin (PIP) (27.00%), trimethoprim/sulfamethoxazole (SXT) (25.50%), and chloramphenicol (C) (19.00%). Isolates were more susceptible to aztreonam (ATM) (94.00%), amoxicillin/clavulanate (AMC) (96.00%), ceftazidime (CAZ) (98.00%), and amikacin (AN) (99.00%). No isolates were resistant to imipenem (IPM) or meropenem (MEM). Additionally, 48 isolates (24.00%) exhibited multiple resistances, and six isolates were found to be resistant to all classes of antibiotics. Notably, 75.51% multiple resistant *E. coli* (37/49) in this study was resistant to tetracycline and β-lactams concurrently. The antibiotic resistance profiles of 200 *coli* isolates were shown in Table S1.

### Analysis of antibiotic resistance level of *E. coli* in two water sources over the dry/wet seasons

**Figure 3:**
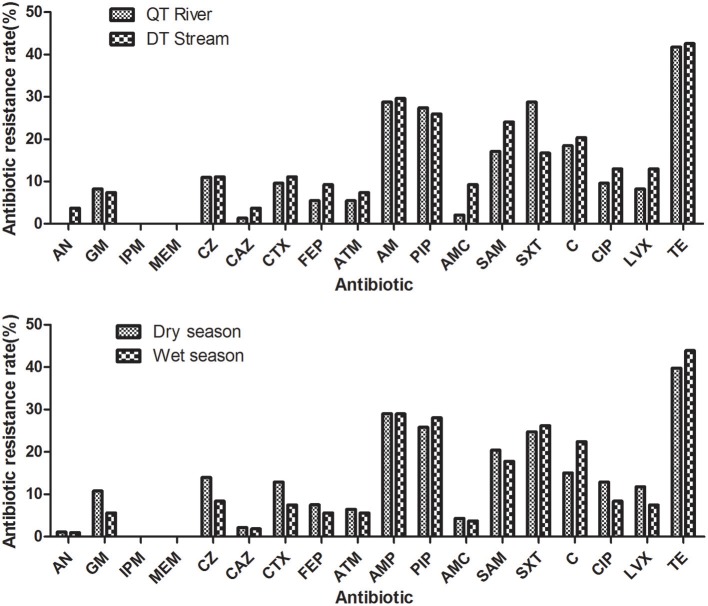
Antibiotic resistance rates of the *E. coli* isolated from the Qiantang River and the Dongtiao Stream over two seasons. AN, amikacin; GM, gentamicin; IPM, imipenem; MEM, meropenem; CZ, cefazolin; CAZ, ceftazidime; CTX, cefotaxime; FEP, cefepime; ATM, aztreonam; AMP, ampicillin; PIP, piperacillin; AMC, amoxicillin/clavulanate; SAM, ampicillin/sulbactam; C, chloramphenicol; CIP, ciprofloxacin; LVX, levofloxacin; TE, tetracycline.

The antibiotic resistance level in different seasons and different water sources was presented in Table 3 and Figure 3. In general, *E.coli* isolated from the Qiantang River and the Dongtiao Stream had similar antibiotic resistant rates. And the dry/wet seasons had minor effects on the resistance rate of *E. coli*. Even for the individual antibiotics tested, still no significant differences can be observed between the dry/wet seasons or between the Qiantang River and the Dongtiao Stream. However, it should be noted that 83.33% isolates (5/6,) resistant to six classes of antibiotics were found in the dry season.

**Table 3:** Antibiotic resistance of *E. coli* isolated from the Qiantang River and the Dongtiao Stream over two seasons.

**Sources**	**No. of *E. coli* isolates**	**No. (%) of R isolates^a^**	**No. (%) of MDR isolates^b^**	**ARI**	**Seasons**	**No. of *E. coli* isolates**	**No. (%) of R isolates^a^**	**No. (%) of MDR isolates^b^**	**ARI**
The Qiantang River	146	71(48.63)	36(24.66)	0.12	dry season	93	45(48.39)	21(22.58)	0.13
The Dongtiao Stream	54	28(51.85)	12(22.22)	0.14	Wet season	107	54(50.47)	27(25.23)	0.12
Total	200	99(49.50)	49(24.50)	0.13		200	99(49.50)	49(24.50)	0.13

a *Resistant E. coli isolates*.

b *MDR isolates, multi-drug resistant E. coli isolates*.

As shown in Table 4, the resistance rate of *E. coli* noticeably decreased (except R1-2 subpopulation in the wet season) from up- to downstream of the Qiantang River. This finding indicates that antibiotic resistance level of *E. coli* can be reduced gradually in source water protection area.

**Table 4:** The distribution of antibiotic resistance rate for R1-2 and R3-6 subpopulation isolated from the Qiantang River in two seasons.

**Sites**	**Dry season**			**Wet season**		
	**No. of *E. coli* isolates analyzed**	**No.(%)of R1-2 isolates^a^**	**No.(%)of R3-6 isolates^b^**	**No. of *E. coli* isolates analyzed**	**No.(%)of R1-2 isolates^a^**	**No.(%)of R3-6 isolates^b^**
Q1	11	4 (36.36)	4 (36.36)	17	3 (17.65)	10 (58.82)
Q2	16	5 (31.25)	7 (43.75)	22	5 (22.72)	3 (13.64)
Q3	17	4 (23.53)	3 (17.64)	19	4 (21.05)	5 (26.32)
Q4	17	0 (0.00)	0 (0.00)	27	9 (33.33)	3 (11.11)
*p*-vaule		0.030	0.005		0.268	0.004

a *Resistance to 1-2 classes of antibiotics*.

b *Resistance to 3-6 classes of antibiotics*.

### Correlation between the antibiotic resistance and water quality parameters in drinking water sources

Spearman's rank correlation analyses were conducted to reveal the correlations between the resistance rates of *E. coli* and water quality parameters in drinking water sources. As shown in Table 5, the results demonstrated that water quality parameters, including the density of *E. coli* and fecal coliforms, did not have correlation with antibiotic resistance rates and multi-drug resistance rates. However, ammonia nitrogen correlated significantly with the resistance rates for gentamicin (GM), cefazolin (CZ), ampicillin/sulbactam (SAM), levofloxacin (LVX) (*p* < 0.05), also a strong but statistically insignificant trend with cefotaxime (CTX), C, and CIP resistance (0.05 < *p* < 0.1). Total phosphorus was closely correlated with the resistance rates for GM (*p* < 0.001), CZ, CTX, and SXT (*p* < 0.05). Total nitrogen was significantly correlated with cefepime (FEP) resistance (*p* < 0.05), while biological oxygen demand (BOD_5_) was correlated with the resistance rate for amikacin (AN) (*p* < 0.05).

**Table 5:** Spearman's rank correlation coefficients between antibiotic resistance rates of *E. coli* and water quality parameters.

	**AR rate**	**MDR rate**	**Aminoglycosydes**		**Beta-lactams**									**Sulphonamides**	**Phenicols**	**Quinolones**		**Tetracy-clines**
			**AN**	**GM**	**Cephalosporins**					**Penicillins**				**SXT**	**C**	**CIP**	**LVX**	**TE**
					**CZ**	**CAZ**	**CTX**	**FEP**	**ATM**	**AM**	**PIP**	**AMC**	**SAM**					
pH	−0.109	−0.085	−0.078	0.037	−0.148	−0.233	−0.185	−0.031	−0.329	−0.055	−0.140	−0.404	−0.070	−0.271	−0.450	−0.466	−0.382	−0.255
Water temp(°C)	−0.213	−0.347	−0.304	−0.537	−0.443	−0.271	−0.394	−0.389	−0.185	−0.097	0.012	−0.425	−0.439	−0.107	−0.304	−0.381	−0.498	0.000
DO(mg/l)	0.164	0.224	0.017	−0.134	−0.104	0.143	−0.141	−0.166	0.206	0.079	−0.030	0.096	0.012	0.134	−0.018	−0.182	−0.183	−0.224
Ammonia nitrogen (mg/l)	0.527	0.612	0.130	0.754^*^	0.669^*^	0.437	0.595	0.499	0.450	0.455	0.370	0.444	0.644^*^	0.535	0.552	0.614	0.665^*^	0.248
Total phosphorus (mg/l)	0.418	0.479	−0.355	0.863^**^	0.706^*^	0.198	0.632^*^	0.474	0.388	0.455	0.430	−0.075	0.395	0.754^*^	0.430	0.511	0.463	0.248
Total nitrogen (mg/l)	0.164	0.321	0.623	0.590	0.411	0.253	0.460	0.671^*^	0.250	0.030	−0.055	0.423	0.353	0.067	0.430	0.511	0.598	0.176
Nitrate nitrogen (mg/l)	−0.170	−0.024	0.304	0.256	0.074	0.219	0.037	0.167	−0.116	−0.249	−0.359	0.253	0.052	−0.262	−0.085	0.024	0.116	−0.334
BOD5 (mg/l)	−0.201	−0.159	0.666^*^	−0.419	−0.525	0.223	−0.426	−0.108	0.145	−0.372	−0.427	0.323	−0.333	−0.358	−0.061	−0.080	−0.113	−0.238
COD (mg/l)	−0.115	−0.030	0.225	0.292	0.104	0.157	0.080	0.209	0.019	−0.200	−0.297	0.116	0.000	−0.213	−0.091	0.097	0.152	−0.309
Fecal coliforms (CFU/100 ml)	−0.212	−0.370	−0.052	−0.596	−0.509	0.102	−0.472	−0.449	0.044	−0.200	−0.115	−0.014	−0.450	−0.207	−0.224	−0.146	−0.293	−0.164
*E. coli* (CFU/100 ml)	−0.049	−0.170	0.390	−0.494	−0.443	0.086	−0.338	−0.139	0.166	−0.109	−0.061	0.140	−0.256	−0.238	−0.024	−0.006	−0.089	0.067

*Bold indicates the relationships are significant statistically*.

* *0.01 ≤ p < 0.05*.

** *0.001 ≤ p < 0.01*.

The association between antibiotic resistance phenotypes of *E. coli* isolates from drinking water sources were analyzed by Pearson's chi-square tests (Table 6). Besides the co-resistance to the same class of antibiotics, resistance to quinolones (CIP and LVX) was closely associated with cephalosporins or GM (*p* < 0.001). In addition, resistance to tetracyclines was associated with CIP resistance (0.001 ≤ *p* < 0.01), and to a relatively weaker extent with phenicols (0.01 ≤ *p* < 0.05).

**Table 6:** Association between antibiotic resistance phenotypes of *E. coli* isolates from drinking water sources.

			**Aminoglycosydes**		**Beta-lactams**									**Sulphonamides**	**Phenicols**	**Quinolones**	
			**AN**	**GM**	**Cephalosporins**					**Penicillins**				**SXT**	**C**	**CIP**	**LVX**
					**CZ**	**CAZ**	**CTX**	**FEP**	**ATM**	**AM**	**PIP**	**AMC**	**SAM**				
Aminoglycosydes		AN															
		GM	+														
Beta-lactams	Cephalosporins	CZ	–	–													
		CAZ	–	–	++												
		CTX	–	+	+++	++											
		FEP	+	–	+++	+++	+++										
		ATM	+	–	+++	+++	+++	+++									
	Penicillins	AM	–	–	+++	–	+++	+	+								
		PIP	–	–	+++	–	+++	++	++	+++							
		AMC	++	–	+++	+	++	+	+	-	+						
		SAM	–	–	–	+	+	-	+	+++	+++	+					
Sulphonamides		SXT	–	–	–	–	–	–	–	–	–	–	–				
Phenicols		C	–	–	–	–	–	–	+	–	–	–	–	–			
Quinolones		CIP	–	+++	+++	+	+++	+++	+++	–	–	–	–	–	–		
		LVX	–	+++	+++	+	+++	+++	+++	–	+	–	+	–	–	+++	
Tetracyclines		TE	–	–	–	–	–	–	–	–	–	–	–	–	+	++	–

*The levels of significance of the association as assessed by pearson's chi-square test: –, p ≥ 0.05; +, 0.01 ≤ p < 0.05; ++, 0.001 ≤ p < 0.01; +++, p < 0.001*.

### Detection of tetracycline resistance genes

Tetracycline was the most prevalent antibiotic resistance in this study, thus five tetracycline resistance genes (*tetA, tetC, tetD, tetE*, and *tetM*) were analyzed in the 82 tetracycline positive *E. coli* isolates (Table S2). Of the five genes examined, *tetA* and *tetC* were the predominant genes. The detection ratios of these genes were: 89.02% for *tetC* (73/82), 70.73% for *tetA* (58/82), 39.02% for *tetD* (32/82), 14.63% for *tetE* (12/82), 9.76% for *tetM* (8/82), respectively. Most of the isolates (81.87%) contained two or more *tet* genes, while *tetD* and *tetM* were detected when other *tet* genes were present. There were 14 different combinations for the *tet* genes: the highest occurrence was *tetA-tetC* (34.15%), followed by *tetA-tetC-tetD* (15.85%), and *tetC-tetD* (7.32%). Some other combinations such as *tetA-tetD* and *tetC-tetE*, were only observed in one to three isolates.

## Discussion

The Polluting of drinking water sources and the presence of antibiotic resistant bacteria increase the risk to human health. It is important to have detailed information regarding such issues. In the present study, we assessed the water quality and the prevalence of antibiotic resistant *E. coli* in two drinking water sources (the Qiantang River and the Dongtiao Stream) in Hangzhou city. Although there was some degree of organic and fecal pollution found in these two sources, the situation was better than those reported at the other sites (Pereira et al., 2013; Ramirez Castillo et al., 2013), which could be the result of the good management of the special water function zone. The influx of fecal pollution deteriorated in the wet season, which was the opposite of organic pollution. This may have been due to rainfall. In the wet season, the feces of domestic animals and contaminated soils are easily washed into rivers by rain, and lead to the rise of bacteriological values. On the contrary, the absence of rain in the dry season reduces the dilution effect of water for pollution, which could cause a higher level of organic pollution. Except for DO and fecal coliforms at site Q4 during the wet season, the physicochemical and bacteriological parameters of all samples met the requirements for Surface Water Environmental Quality Standard for grade III (GB3838-2002).

In contrast to other types of pollution, antibiotic resistance is difficult to remove even if the release of antibiotic resistance determinants in the environment is discontinued (Martinez, 2009). In this study, 49.50% of the *E. coli* isolated was resistant to at least one of the tested antibiotics (with an ARI of 0.12) and 24.00% exhibited multiple resistances. The levels of antibiotic resistance are comparable to that in other aquatic environments (Chen et al., 2011; Ribeiro et al., 2012; Machado and Bordalo, 2014; Zhang et al., 2014). It indicates the issue of antibiotic resistance is also serious in qualitied source water. Most *E. coli* isolates detected here were resistant to tetracycline, followed by β-lactams (AMP and PIP), sulphonamides, and chloramphenicol, which is consistent with other studies (Maal-Bared et al., 2013; Pereira et al., 2013). This was expected as these antibiotics were old and they have been widely used in human and veterinary medicine (Jiang et al., 2013; Maal-Bared et al., 2013; Pereira et al., 2013). In Zhejiang province, tetracycline and chloramphenicol are the principal antibiotics used for livestock (Cui, 2011), and sulphonamides were also frequently detected in wastewater from piggeries and duck farms (Wu et al., 2012). Notably, 75.51% of multiple resistant *E. coli* (37/49) in this study was resistant to tetracycline and β-lactams concurrently. *E. coli* isolates from WWTP effluents or from the aquatic environments contaminated by these effluents, are present consistently resistant to tetracycline and β-lactams, and are associated with at least one other resistance, such as sulfamides, chloramphenicol, quinolones, in any combination (Ribeiro et al., 2012). Thus, a likely hypothesis for the origin of these multiresistant isolates is the WWTP effluents.

Thankfully, the resistance rate of *E. coli* noticeably decreased (with the exception of the R1-2 subpopulation in the wet season) from up- to downstream in the Qiantang River. This phenomenon, however, seems to contradict other reports (Ham et al., 2012; Zhang et al., 2014). Special water function zones with strict safeguards are possibly the main cause of such paradoxical phenomenon (Tao et al., 2010). In China, the range of secondary source water protection areas are not less than 3 km from intake upstream sites and not less than 300 m from the intake downstream locations. And Water Sources Protection Zones for Pollution Prevention and Control Regulations clearly point out that waste water, garbage, and any other activities that can cause water pollution are prohibited (not excluding unmanageably scattered pollutions) in water source protection areas. Thus, few new pollutants are discharged into such areas, and the level of antibiotic resistant *E. coli* from upstream decreases gradually due to gene loss or environmental self-purification.

Seasonal variation is a possible factor for the change of antibiotic resistance (Mohanta and Goel, 2014). For example, the percentage of multi-drug resistant (MDR) bacteria from three different water sources in West Bengal followed the trend: post-monsoon>winter>summer (Mohanta and Goel, 2014). Similarly, the number of antibiotic resistant isolates from wells in West Africa was higher in the dry season than that in the wet season (Machado and Bordalo, 2014). However, our data indicates that no correlation was found between seasonality and the antibiotic resistance level of *E. coli*, which is consistent with the previous study (Maal-Bared et al., 2013). The discrepancy may be the result of the different major pollution sources and pollution types. For aquatic environments that mainly receives pollutants from scattered sources (such as farmlands and animal farms), leaching and runoff events, which are often seasonal, probably cause the change of antibiotic resistance in different seasons (Laroche et al., 2010). Point source pollution is the main pollution type in the Qiantang River and the Dongtiao Stream. Pollutants from wastewater treatment plants (WWTPs) or large/small enterprises in the upper streams of the rivers are relatively stable all year round, despite the existence of some small scattered pollution sources.

The relationships between certain water quality variables and antibiotic resistance of bacteria or ARGs have been reported by several studies, such as water depth, temperature, river flow, precipitation, ammonium, total dissolved solids concentrations, dissolved oxygen (Maal-Bared et al., 2013; Middleton and Salierno, 2013; Staley et al., 2015). In this study, we analyzed the links of some water quality parameters and the antibiotic resistance of *E. coli*. The resistance to different antibiotics had different associations with water quality parameters. The frequency of *E. coli* resistance to a part of the antibiotics had strong positive associations with nutrient concentrations, which was in accordance with the studies in British Columbia (Maal-Bared et al., 2013) and in northern China (Zhang et al., 2014). Blanco et al. explained that the addition of nutrient concentrations enhanced horizontal transfer of genetic resistance elements (Blanco et al., 2009). *E. coli* isolates carrying genetic resistance elements had much stronger viability than sensitive isolates under high nutrient concentrations, which led to increasing resistance. No relationships existed among water temperature, pH value, or DO with antibiotic resistance, which was different from other studies. Williams et al. (Williams et al., 1996) deemed that higher temperatures were also the stimulant for the increasing of natural transformation rates among microorganisms. Alkaline conditions easily made antibiotics degrade and hence decreased the resistance (Doi, 2000; Maal-Bared et al., 2013). The discrepancies with these studies are possibly due to the fact that all of the pH values in our study are neutral and the variable range (7.31–7.66) is negligible. For water temperature, even though the change range (14.2–22.7°C) is large, the lowest temperature is suitable for the growth and activity of *E. coli*. Water quality parameters were measured instantaneously, and hardly exhibit any direct effect on antibiotic resistance which is the result of prolonged period. Moreover, many important factors (such as suspended particles and biofilm) in aquatic environments, can not only influence the development of antibiotic resistance, but can also be influenced by water quality parameters (Dang and Lovell, 2016). All of these relative factors in the Qiantang River and the Dongtiao Stream have not been considered. Furthermore, natural resistance in the *E. coli* population present in water sources has also not been considered. Thus, these results should still be explained with caution.

Significant correlations were found frequently among the resistance rates of different antibiotics, and the correlations among tetracycline, chlortetracycline, and quinolone resistance were reported the most (Maal-Bared et al., 2013; Zhang et al., 2014). Similarly, in this study, we also found significant correlations between resistance to tetracycline and chloramphenicol or CIP. These strong correlations are probably the results of co-selection (Dang et al., 2006). In fact, tetracycline efflux proteins have similar amino acid and protein structure with other efflux proteins, including chloramphenicol and quinolone resistence, which result the concurrent resistance to multiple antibiotics (Chopra and Roberts, 2001). Besides, resistance to quinolone (CIP and LVX) also had significant correlations with resistance to cephalosporin or GM. They may indicate that an analogous mechanism of cross-selection and/or co-selection among these antibiotics had occured as well (Courvalin and Trieu-Cuot, 2001; Dang et al., 2006; Zhang et al., 2014). Moreover, many antibiotic resistance genes are often found on the same plasmid or mobile genetic elements (Chopra and Roberts, 2001; Roberts, 2005; Carattoli, 2013), which results in the correlations found among resistance to different antibiotics. The *StrA-strB* gene pair and the *sul2* or *tet* genes were found in the same plasmid acquired in multiple resistant bacteria from the surface seawater of Jiaozhou Bay (Zhao and Dang, 2011). *TetQ* is often associated with a large conjugative transposon which carries the *ermF* (Chopra and Roberts, 2001). Thus, further studies should be carried out to illustrate the exact reasons of these correlations.

The frequent detection of tetracycline resistance genes have been reported widely in aquatic environments (Tao et al., 2010; Jia et al., 2014; Chen et al., 2015). In this study, tetracycline resistance also shows the highest resistance frequency among all of the antibiotics tested. Thus, we analyzed five tetracycline resistance genes (*tetA, tetC, tetD, tetE*, and *tetM*) from 82 tetracycline resistant *E. coli* isolates. As the representatives of efflux mechanism of tetracycline, *tet*A and *tet*C was widely found in gram-positive and gram-negative bacteria (Zhang et al., 2009). Similarly, our study also showed *tetA* and *tetC* were predominant genes (89.02 and 70.73%, respectively) in water sources. This may suggest the risk for increasing levels of multiple resistant bacteria, because these efflux genes are normally associated with large plasmids, and these plasmids often carry other antibiotic resistance genes (Chopra and Roberts, 2001). *TetM*, a gene for encoding ribosomal protection protein, had the lowest detectablerate. This result supports the fact that some gram-positive *tet* genes, such as *tetK, tetL, tetO, tetM*, are not often examined in gram-negative bacteria. In addition, we found it is common for most resistant *E. coli* isolates (81.87%) to carry two or more *tet* genes. Interestingly, all of the *tetD* and *tetM* were present with other *tet* genes, while *tetA, tetC* and *tetE* were sometimes the sole detectable *tet* genes. They are totally consistent with the study in Australia (Akinbowale et al., 2007).

## Conclusions

The quality of drinking water sources detected in Hangzhou city conformed to the Surface Water Environmental Quality Standard of China, which deems these water sources safe enough to supply to water plants. Despite this, antibiotic resistant *E. coli* isolates were still heavily seen in these source waters. As antibiotic resistance is hard to eliminate and *E. coli* is proficient at horizontal transfer, the prevalence of antibiotic resistance *E. coli* in source water reveals a potential public health risk. Thanks to the special water function zones that offer protection reducing new pollutants, the resistance rates of *E. coli* decreased gradually from upstream to downstream in Qiantang River. It indicates controlling pollutant emissions and anthropogenic activities could be an effective method to lower the level of antibiotic resistance. Seasonality (dry and wet seasons) had no effect on the resistance rates of *E. coli* isolates. While some water quality parameters (ammonia nitrogen, total phosphorus, total nitrogen, and BOD_5_) showed strong relationships with antibiotic resistance that is worth studying further. In addition, significant correlations were found among the resistance rates of different antibiotics. The specific reasons for this should also be explored further.

Because of the highest resistance to tetracycline, five *tet* genes (*tetA, tetC, tetD, tetE, and tetM)* were detected from tetracycline resistant positive isolates. We found that *tetA* and *tetC* were the predominant genes in source water. Considering tetracycline efflux genes are usually associated with large plasmids, and these plasmids often carry other antibiotic resistance genes, the potential risk for increasing multiple resistance bacteria in drinking water sources should be attached great importance.

## Author contributions

ZC performed the isolation of *E. coli* and part of antibiotic susceptibility test, all of data analysis, and wrote the manuscript. DY supervised all the experiments. SH performed part of antibiotic susceptibility test. HY drew the map and measured part of water quality parameters. YW detected all tetracycline resistance genes. WZ and LS collected water samples and measured part of water quality parameters. LZ and SC designed the study.

### Conflict of interest statement

The authors declare that the research was conducted in the absence of any commercial or financial relationships that could be construed as a potential conflict of interest.

## Abbreviations

ARB: antibiotic resistant bacteria
ARGs: antibiotic resistance genes
*E. coli*: *Escherichia coli*
MDR: multi-drug resistant
ARI: Antibiotic resistance index
AN: amikacin
GM: gentamicin
IPM: imipenem
MEM: meropenem
CZ: cefazolin
CAZ: ceftazidime
CTX: cefotaxime
FEP: cefepime
ATM: aztreonam
AMP: ampicillin
PIP: piperacillin
AMC: amoxicillin/clavulanate
SAM: ampicillin/sulbactam
C: chloramphenicol
CIP: ciprofloxacin
LVX: levofloxacin
TE: tetracycline
DO: dissolved oxygen
Water temp.: water temperature
COD: Chemical oxygen demand
N0^3−^-N: Nitrate-N
TP: total phosphorus
TN: total nitrogen.
